# Frail phenotype might herald bone health worsening among end-stage renal disease patients

**DOI:** 10.7717/peerj.3542

**Published:** 2017-07-10

**Authors:** Chia-Ter Chao, Jenq-Wen Huang, Ding-Cheng Chan, Cohort of Geriatric Nephrology in NTUH (COGENT) study group

**Affiliations:** 1 Department of Medicine, National Taiwan University Hospital Jinshan branch, New Taipei City, Taiwan; 2 Department of Internal Medicine, National Taiwan University Hospital, Taipei, Taiwan; 3 Graduate Institute of Toxicology, National Taiwan University, Taipei, Taiwan; 4 Department of Geriatrics and Gerontology, National Taiwan University Hospital, Taipei, Taiwan

**Keywords:** Bone mass, Osteoporosis, Dialysis, Dual energy X-ray absorptiometry, Frailty, End-stage renal disease, Chronic kidney disease

## Abstract

**Background:**

Frailty exhibits a high prevalence in end-stage renal disease (ESRD) patients and is associated with adverse health-related outcomes, including falls and fractures. Available studies do not address whether frailty is associated with temporal changes in BMD. We evaluated this issue by analyzing the follow-up dual energy X-ray absorptiometry (DXA) results in an ESRD cohort.

**Methods:**

In 2015, we enrolled forty-three ESRD patients, divided into frail, pre-frail, and robust ones based on a validated simple FRAIL scale, all receiving DXA at baseline. After one year of follow-up, survivors received another DXA, and we calculated the absolute and percentage changes in area, bone mineral density (BMD), *T*-, and *Z*-scores of lumbar spine and femoral neck (FN) between baseline and follow-up examinations.

**Results:**

Among all, frail individuals with ESRD had significantly lower average lumbar spine area, lower L4, FN, and total BMD and *T*-scores, lower FN and total *Z*-scores than non-frail ones, without differences in gender, body mass index, dialysis duration, and comorbidities. Furthermore, we discovered frail ESRD patients had significantly more prominent decrease in average lumbar spine area, percentage changes in L1 *Z*-scores and average lumbar spine area, and a trend toward more prominent decrease in L4 area than non-frail ones after one year of follow-up.

**Conclusions:**

Baseline frailty might be associated with deteriorating bone health, including shrinking *L*-spine areas and a more rapid decrease in *L*-spine *Z* scores, among ESRD patients. This frailty-bone association should be highlighted during our care of frail individuals with ESRD.

## Introduction

Frailty exhibits a high prevalence in end-stage renal disease (ESRD) patients and similarly predicts adverse health-related outcomes as it does in older adults. Self-reported frailty has been suggested to predict falls and fractures among ESRD patients (Delgado et al., 2015), raising the possibility that frailty can impact bone mass. Hospitalized older adults with frailty were found to have a significantly higher risk of prospective fall episodes than robust ones (Joseph et al., 2017), lending support to this theory. Indeed, existing studies already discovered that the presence of frailty was associated with lower bone mineral density (BMD) in lumbar vertebrae or femoral neck (FN) in older adults (Liu et al., 2015; Cook et al., 2016), and we derived similar findings in an ESRD cohort previously (Chao et al., 2016b). However, available studies are essentially cross-sectional in nature, and the relationship between frailty and temporal changes in BMD, especially among ESRD patients, is still unclear. As a pilot attempt, we evaluated this issue by analyzing the follow-up dual energy X-ray absorptiometry (DXA) results in our original ESRD cohort.

## Methods

The current study has been approved by the ethical review board of National Taiwan University Hospital (NO. 201505154RINB), and the study protocol was described previously (Chao et al., 2016b; Chao et al., 2016a; Chao et al., 2016c; Chao, Chan & Huang, 2017). All patients provided written inform consent.

In 2015, we enrolled forty-three ESRD patients (mean 67.4 ± 9.8 years, 46.5% male) who received chronic hemodialysis, divided into frail (14%), pre-frail (51%) and robust (35%) ones based on a validated simple FRAIL scale (SFS; higher scores indicating more severe frailty) (Chao et al., 2015; Chao et al., 2016b), all receiving DXA at baseline. The results of their DXA findings are available elsewhere (Chao et al., 2016b). After one year of follow-up, seven (16.3%) died, and the remaining thirty-six (83.7%) received another DXA in standard positions as they did one year ago. We calculated the absolute and percentage changes in area, BMD, *T*-, and *Z*-scores of lumbar spine and FN between baseline and follow-up examinations 1 year later, and analyzed the correlation between the 2016 DXA parameters as well as changes with SFS scores as a continuous variable, an estimate of frail severity, using Pearson’s correlation. Finally, we compared the clinical profiles and the above DXA parameters between those with and without frailty during the initial assessment using a Student’s *t*-test, followed by univariate and multivariate analyses through multiple linear regression, to discern the relationship between frailty and temporal changes in bone mineral and morphological parameters.

## Results

Among all ESRD survivors (mean 69.1 ± 9.1 years, 47.2% male; frail 14%, pre-frail 53%, robust 33%), the mean body mass index (BMI) was 22.8 ± 3.2. The BMI among those with and without frailty were 22.6 ± 1.8 and 22.8 ± 3.4, respectively, without significant difference (*p* = 0.88). The clinical features of the survivors, including comorbidities and laboratory data, were provided in Table 1. Frail survivors with ESRD did not differ significantly from non-frail ones with regard to dialysis duration, most comorbidities and laboratory parameters examined, except serum albumin and creatinine.

**Table 1:** Clinical features of the enrolled ESRD survivors, after 1-year of follow-up.

	Total	Frail	Non-frail	*p value*
*Vintage*	*3.5 ± 2.9*	*4.4 ± 3.8*	*3.3 ± 2.8*	*0.48*
*Comorbidity*				
Diabetes mellitus	17 (47)	4 (80)	13 (42)	*0.12*
Hypertension	31 (86)	4 (80)	27 (87)	*0.68*
Heart failure	6 (17)	1 (20)	5 (16)	*0.84*
Cirrhosis	4 (11)	1 (20)	3 (10)	*0.51*
Cancer	4 (11)	1 (20)	3 (10)	*0.51*
Thyroid illness	4 (11)	1 (20)	3 (10)	*0.51*
Rheumatologic illness	2 (6)	0 (0)	2 (6)	*0.57*
*Laboratory profile*				
Albumin (g/dL)	3.8 ± 0.3	3.5 ± 0.3	3.9 ± 0.3	*0.02*
Urea nitrogen (mg/dL)	81.9 ± 18.9	75.1 ± 16.9	82.9 ± 19.3	*0.4*
Creatinine (mg/dL)	11 ± 2.2	8.7 ± 0.6	11.4 ± 2.1	*<0.01*
Potassium (meq/L)	4.8 ± 0.7	4.4 ± 0.6	4.8 ± 0.7	*0.18*
Calcium (mg/dL)	9 ± 0.8	9 ± 0.9	9.1 ± 0.8	*0.94*
Phosphate (mg/dL)	5.2 ± 1.6	4.2 ± 1.5	5.3 ± 1.5	*0.15*
Alkaline phosphatese (U/L)	75.6 ± 31.7	96.4 ± 24.7	72.2 ± 31.7	*0.12*
Intact parathyroid hormone (pg/ml)	345 ± 285	299 ± 181	352 ± 300	*0.71*
Hemoglobin (mg/dL)	9.7 ± 1.3	8.8 ± 1.8	9.8 ± 1.2	*0.09*
Leukocyte (K/µL)	7.1 ± 2.7	8.9 ± 3.5	6.8 ± 2.5	*0.11*
Platelet (K/µL)	199 ± 61	204 ± 82	199 ± 58	*0.86*

**Notes.**

ESRD end-stage renal disease

Survivors with frailty at baseline had significantly lower average lumbar spine area (*p* < 0.01), lower L4 (*p* = 0.04), FN (*p* < 0.01), and total (*p* < 0.01) BMD and *T*-scores, lower FN (*p* = 0.03) and total (*p* < 0.01) *Z*-scores than non-frail ones (Table 2), but without differences in gender (frail vs. non-frail, 20% vs. 52%, *p* = 0.2), BMI (frail vs. non-frail, 22.6 ± 1.8 vs. 22.8 ±  3.4 kg/m^2^, *p* = 0.88), dialysis duration (frail vs. non-frail, 4.4 ± 3.8 vs. 2.2 ± 2.8 years, *p* = 0.48), comorbidities including diabetes mellitus (*p* = 0.12), hypertension (*p* = 0.68), and heart failure (*p* = 0.84). Similarly, there were no differences between frail and non-frail ESRD survivors regarding serum albumin (*p* = 0.08), urea nitrogen (*p* = 0.4), potassium (*p* = 0.18), calcium (*p* = 0.94), phosphate (*p* = 0.15), hemoglobin (*p* = 0.26), and total cholesterol (*p* = 0.5), except higher creatinine (*p* < 0.01) among the latter group.

**Table 2:** Findings from follow-up DXA examinations among the enrolled ESRD survivors.

	Frail	Non-frail	*p value*
*Area (cm*^2^*)*			
**L1**	13 ± 1	14.6 ± 1.9	*0.17*
Change	−0.27 ± 1.67	0.44 ± 1.66	*0.48*
Change %	−1.62 ± 12.1	3.81 ± 11	*0.43*
**L2**	14.3 ± 1.7	14.7 ± 1.8	*0.72*
Change	0.75 ± 2.76	0.06 ± 0.81	*0.29*
Change %	6.89 ± 21	0.57 ± 5.53	*0.18*
**L3**	15.8 ± 3.2	16.1 ± 2.4	*0.83*
Change	0.47 ± 4.31	0.05 ± 2.3	*0.74*
Change %	105.5 ± 32.9	101.7 ± 20.3	*0.73*
**L4**	17 ± 2.6	17.9 ± 2.7	*0.56*
Change	−1.51 ± 4.27	0.91 ± 2.07	***0.08***
Change %	−4.04 ± 21.3	6.2 ± 12.4	*0.18*
**L-spine average**	45.8 ± 13.4	59.5 ± 9.2	***<0.01***
Change	−9.97 ± 11.1	3.91 ± 6.18	***<0.01***
Change %	−18.3 ± 21.2	8.07 ± 12.4	***<0.01***
**Femoral neck**	4.88 ± 0.27	5.06 ± 0.5	*0.44*
Change	−0.09 ± 0.54	0.13 ± 0.78	*0.54*
Change %	−0.7 ± 11.3	12.9 ± 70.6	*0.67*
**Total**	31.7 ± 4.1	35.8 ± 6.4	*0.18*
Change	−0.72 ± 3.84	2.09 ± 4.25	*0.18*
Change %	−1.88 ± 11.67	6.27 ± 13.3	*0.2*
*Bone mineral density, g/cm*^2^			
**L1**	0.76 ± 0.07	0.92 ± 0.15	***0.07***
Change	<0.01 ± 0.05	0.02 ± 0.05	*0.64*
Change %	1.65 ± 7.47	3.05 ± 7.14	*0.75*
**L2**	0.92 ± 0.12	0.93 ± 0.17	*0.94*
Change	0.06 ± 0.23	0.02 ± 0.05	*0.38*
Change %	9.9 ± 30.5	2.09 ± 6.06	*0.2*
**L3**	0.85 ± 0.14	0.98 ± 0.19	*0.16*
Change	0.02 ± 0.06	−0.03 ± 0.21	*0.62*
Change %	2.92 ± 7.34	−2.06 ± 18.9	*0.57*
**L4**	0.73 ± 0.12	0.95 ± 0.19	***0.04***
Change	−0.01 ± 0.1	0.01 ± 0.05	*0.45*
Change %	−0.83 ± 13.4	1.59 ± 5.22	*0.51*
**L-spine average**	0.83 ± 0.11	0.95 ± 0.17	*0.14*
Change	0.02 ± 0.07	0.02 ± 0.03	*0.77*
Change %	2.64 ± 9.95	2.48 ± 4.02	*0.95*
**Femoral neck**	0.45 ± 0.09	0.64 ± 0.12	***<0.01***
Change	<0.01 ± 0.05	<0.01 ± 0.04	*0.91*
Change %	2.38 ± 12.1	1.18 ± 6.07	*0.73*
**Total**	0.54 ± 0.14	0.78 ± 0.15	***<0.01***
Change	<-0.01 ± 0.03	<0.01 ± 0.05	*0.83*
Change %	0.51 ± 7.37	0.89 ± 7.5	*0.92*
*T-score*			
**L1**	−1.83 ± 0.57	−0.47 ± 1.21	***0.07***
Change	0.07 ± 0.47	0.22 ± 0.43	*0.56*
Change %	−4.93 ± 28.2	9.65 ± 160.1	*0.88*
**L2**	−0.57 ± 1.06	−0.54 ± 1.41	*0.97*
Change	0.5 ± 1.99	0.15 ± 0.44	*0.39*
Change %	−57.1 ± 175	8.65 ± 62.4	*0.16*
**L3**	−1.62 ± 1.18	−0.55 ± 1.6	*0.16*
Change	0.3 ± 0.62	0.14 ± 0.3	*0.45*
Change %	43.7 ± 57.2	24.6 ± 113	*0.78*
**L4**	0.73 ± 0.12	0.95 ± 0.19	***0.04***
Change	−0.01 ± 0.1	0.01 ± 0.05	*0.51*
Change %	−0.83 ± 13.4	1.59 ± 5.22	*0.45*
**L-spine average**	−1.6 ± 0.96	−0.56 ± 1.4	*0.12*
Change	0.04 ± 0.63	0.17 ± 0.32	*0.5*
Change %	−22.3 ± 62.1	33.7 ± 105	*0.26*
**Femoral neck**	−3.34 ± 0.76	−1.63 ± 1.02	***<0.01***
Change	0.04 ± 0.43	0.06 ± 0.36	*0.92*
Change %	−0.56 ± 12	2.29 ± 77.6	*0.94*
**Total**	−2.84 ± 1.04	−0.97 ± 1	***<0.01***
Change	−0.02 ± 0.28	0.06 ± 0.4	*0.67*
Change %	−2.27 ± 8.49	3.78 ± 72.2	*0.86*
*Z-score*			
**L1**	0.13 ± 0.32	0.67 ± 0.73	*0.22*
Change	0.1 ± 0.35	0.22 ± 0.34	*0.58*
Change %	−33.3 ± 115	38.1 ± 53.4	***0.05***
**L2**	1.27 ± 1.47	0.75 ± 0.79	*0.32*
Change	0.43 ± 1.45	0.17 ± 0.34	*0.4*
Change %	126 ± 516	29.6 ± 110	*0.38*
**L3**	0.68 ± 1.06	0.81 ± 1.12	*0.81*
Change	0.2 ± 0.35	0.26 ± 0.63	*0.86*
Change %	48.6 ± 86.9	170 ± 627	*0.71*
**L4**	0 ± 0.42	0.72 ± 1.08	*0.2*
Change	−0.05 ± 0.61	0.1 ± 0.3	*0.45*
Change %	100 ± 408	61 ± 114	*0.69*
**L-spine average**	0.64 ± 0.86	0.74 ± 0.89	*0.81*
Change	0.04 ± 0.44	0.18 ± 0.23	*0.3*
Change %	103 ± 294	76.1 ± 131	*0.74*
**Femoral neck**	−0.98 ± 0.76	0.02 ± 0.9	***0.03***
Change	0.14 ± 0.48	0.14 ± 0.42	*0.99*
Change %	0.63 ± 44.8	31.4 ± 98.8	*0.51*
**Total**	−1.5 ± 0.7	0.09 ± 0.87	***<0.01***
Change	0 ± 0.29	0.09 ± 0.41	*0.67*
Change %	−4.03 ± 16.9	−10.3 ± 143	*0.93*

**Notes.**

Abbreviations DXA dual energy X-ray absorptiometry ESRD end stage renal disease

We discovered that frail ESRD survivors had significantly more prominent decrease in average *L*-spine area (*p* < 0.01), percentage changes in L1 *Z*-scores (*p* = 0.05) and average *L*-spine area (*p* < 0.01), and a trend toward more prominent decrease in L4 area (*p* = 0.08) after follow-up than non-frail ones (Table 2). Frailty severity, manifesting as SFS scores, also correlated significantly with L1 (*r* =  − 0.37, *p* = 0.04), L2 (*r* =  − 0.37, *p* = 0.03), L4 (*r* =  − 0.35, *p* = 0.05), average (*r* =  − 0.39, *p* = 0.02), and total (*r* =  − 0.49, *p* < 0.01) lumbar spine areas; L1 (*r* =  − 0.39, *p* = 0.03), L4 (*r* =  − 0.42, *p* = 0.02), average lumbar spine (*r* =  − 0.35, *p* = 0.04), FN (*r* =  − 0.58, *p* < 0.01), and total (*r* =  − 0.61, *p* < 0.01) BMD; L1 (*r* =  − 0.38, *p* = 0.03), L4 (*r* =  − 0.41, *p* = 0.02), average lumbar spine (*r* =  − 0.36, *p* = 0.03), FN (*r* =  − 0.6, *p* < 0.01), and total (*r* =  − 0.6, *p* < 0.01) *T*-scores; FN (*r* =  − 0.44, *p* = 0.01) and total (*r* =  − 0.4, *p* = 0.02) *Z*-scores 1 year later. In addition, SFS scores exhibited significant correlations with changes in L4 (*r* =  − 0.35, *p* = 0.05) and average lumbar spine (*r* =  − 0.45, *p* < 0.01) areas, percentage changes in average lumbar spine areas (*r* =  − 0.41, *p* = 0.01), and exhibited borderline correlations with percentage changes in L1 (*r* =  − 0.33, *p* = 0.08) and L2 *Z*-scores (*r* =  − 0.35, *p* = 0.06), with changes in L4 (*r* =  − 0.33, *p* = 0.09) and total (*r* =  − 0.32, *p* = 0.06) areas one year later.

Finally, we used linear regression analyses with DXA findings as the dependent variables. Continuous variables considered in the regression analyses were all normally distributed, as indicated by the Kolmogorov–Smirnov test results, including age (*p* = 0.2), BMI (*p* = 0.2), dialysis duration (*p* = 0.06), and laboratory data (albumin, urea nitrogen, creatinine, electrolytes, hemoglobin, and total cholesterol). Univariate analyses disclosed that age, BMI, and serum albumin were significantly associated with multiple bone mineral and morphologic parameters, including lumbar spine, FN, and total BMD and *Z*-scores. Serum alkaline phosphatase levels were also significantly associated with changes in L1 BMD/*T*-score, average BMD, and with percentage changes in L4 BMD/*Z*-score, average and total BMD. Intact parathyroid hormone levels were significantly associated with changes in L1 area/BMD/*T*-score/*Z*-score, average BMD, FN *T*-score/*Z*-score, total BMD/*T*-score/*Z*-score, and with percentage changes in L1 area/BMD/*Z*-score, FN BMD/*Z*-score, and total BMD.

In multivariate regression analyses accounting for significant univariate variables (age, BMI, and albumin), we found that baseline frailty was associated with a lower L1 (ß = −0.4, *t* =  − 2.18, *p* = 0.04), L4 (ß = −0.39, *t* =  − 2.1, *p* = 0.046), FN (ß = −0.5, *t* =  − 2.96, *p* < 0.01), total (ß = −0.53, *t* =  − 3.27, *p* < 0.01) BMD, and a more prominent decline of percentage changes in L1 *Z*-score (ß = −0.45, *t* =  − 2.11, *p* = 0.049), average *L*-spine areas (ß = −0.48, *t* =  − 2.84, *p* < 0.01), and in changes of average *L*-spine areas (ß = −0.5, *t* =  − 3.02, *p* < 0.01), one year later. There was no sign of multicollinearity in these models, as demonstrated by the low variance inflation factors (VIFs) (less than five) for all the models above. These associations persisted even after adjusting for serum alkaline phosphatase and intact parathyroid hormone levels.

## Discussion

Since osteoporosis and related fracture confers enormous burden on older adults, ameliorating the risk factors of osteoporosis and incident fractures assumes increasing importance. Risk factors for osteoporosis and poor bone health in older adults include lifestyle issues (physical inactivity, substance abuse), endocrine disorders (androgen insufficiency, hyperparathyroidism), malnutrition, rheumatologic diseases and their associated treatments, neurologic illnesses (Parkinsonism, epilepsy), and various medications (Cosman et al., 2014). The causality between these factors and osteoporosis is often established through the combination of an increased risk of incident fractures conferred by such traits and their concurrent association with osteoporosis. Only part of the identified risk factors exhibit an association with temporal changes in bone mineral parameters, which is a more robust evidence of their biologic influences on osteoporosis. The presence of frailty, an estimate of ageing-associated vulnerability to stressors, has also been shown to increase the risk of incident fractures, through sharing a common panel of risk factors with osteoporosis (Kojima, 2016). However, whether frailty contributes to osteoporosis, or vice versa, remains an open question.

Bone health in patients with chronic kidney disease (CKD) and ESRD is influenced by factors in addition to the traditional risk factors of osteoporosis; renal osteodystrophy, now termed CKD-mineral bone disorder (CKD-MBD), compounds the scenario due to altered divalent ion balances, secondary hyperparathyroidism, vitamin D insufficiency, etc. This pathophysiologic complexity in those with CKD presumably weakens the relationship between low bone mass and the risk of subsequent fracture validated in post-menopausal patients, rendering the diagnosis and treatment of osteoporosis in this population difficult. Patients with CKD-MBD have estimated glomerular filtration rate (eGFR)-dependent impairment in bone volume, mineralization, and turnover (Goldstein, Jamal & Moyses, 2015); results based on bone biopsies from these patients can manifest as osteomalacia, osteoporosis, or adynamic bone disease even when they have similar biochemical indices (Torres et al., 2014). The lack of high quality evidence leads to the recommendation against using DXA to routinely evaluate the risk of fracture in patients with CKD by the 2009 Kidney Disease Improving Global Outcomes guideline (Moe et al., 2009); however, subsequent cohort studies indicate that lower femoral neck or total hip BMD was associated with an elevated risk of fractures in patients with CKD and ESRD (Limori et al., 2012; Yenchek et al., 2012). A meta-analysis also suggested that those with CKD and fracture did have significantly lower femoral, lumbar spine, and radial BMD than those with CKD but without fracture (Bucur et al., 2015). An expert panel revisiting the 2009 KDIGO guideline on CKD-MBD subsequently agreed that the recommendation against using BMD for risk stratification in patients with CKD should be revised (Ketteler et al., 2015), an opinion also supported by others (West, Patel & Jamal, 2015). These findings re-ignite the concept that DXA might still play a role in fracture risk prediction among patients with renal insufficiency. Our results further pinpoint a potential risk factor for altered DXA findings in ESRD patients.

For CKD/ESRD patients, emerging studies revealed that frailty similarly correlates with low bone mass and a higher risk of fractures of vertebrae as well as femur after follow-up (Delgado et al., 2015; Chao et al., 2016b), but it is still unclear whether frailty exerts a biologic effect on temporal changes in BMD among these patients. Although Bleicher et al. (2013) previously reported that frailty was not associated with changes in BMD among older men, their findings might not be able to be extrapolated to the CKD/ESRD population. In this pilot study, we discovered that among ESRD patients, baseline frailty was associated with lower lumbar spine and FN BMD one year later; more importantly, baseline frailty was also associated with worsening of several bone mass and morphologic parameters, including shrinking *L*-spine areas and a more rapid decrease in *L*-spine *Z* scores (Table 2). We propose that the uremic milieu described above, the high prevalence of polypharmacy and potentially inappropriate medications in CKD/ESRD patients, and the extra-skeletal adverse effect brought by frailty such as nutritional impairment (Chao et al., 2015), might modify the relationship between frailty and changes in bone mass over time. More studies are needed to elucidate the mechanisms behind this phenomenon.

Increasing physical activity and dedicated exercise programs have been reported to improve bone health, leading to a lower risk of incident fractures (Liu et al., 2015; Granacher et al., 2013). Since frailty is associated with lower BMD and poorer bone health, interventions focusing on frailty reduction might be an under-recognized way to restore BMD. Multidisciplinary programs against frailty might therefore be recommended for the bone health of frail individuals, especially those with ESRD.

## Limitations

This study was limited in several aspects, the most important of which was the modest sample size. This might limit the statistical efficacy to detect the true relationship between frailty and changes in DXA parameters. As we explained above, this study was a pilot attempt, and studies of larger size are needed to confirm our findings. In addition, only DXA was used to evaluate bone mass in this study, and other image modalities might be needed for better calibration. Finally, we only measured frail severity once at baseline in this study, and a repeated assessment after one year can be more informative with regard to the influence of frailty changes on those of BMD results. Despite these limitation, our findings are still informative for those who caring for frail ESRD patients, and may provide the basis for devising newer treatments for osteoporosis in these patients.

##  Supplemental Information

10.7717/peerj.3542/supp-1 File S1 Supplementary file
